# Social support and mental well-being among people with and without chronic illness during the Covid-19 pandemic: evidence from the longitudinal UCL covid survey

**DOI:** 10.1186/s40359-024-01596-x

**Published:** 2024-03-11

**Authors:** Ozan Aksoy, Alison Fang-Wei Wu, Sevgi Aksoy, Carol Rivas

**Affiliations:** 1 UCL Social Research Institute, University College London, 55-59 Gordon Square, WC1H 0NU London, UK; 2 University of Greenwich, Old Royal Naval College, Park Row, SE10 9LS London, UK

**Keywords:** Chronic illness, Mental wellbeing, Psychological distress, COVID-19, SEM

## Abstract

**Purpose:**

An immediate research priority recovering from the COVID-19 pandemic is well-being among some of our most vulnerable—people with chronic illness. We studied how mental health changed among people with and without chronic illness throughout the pandemic and the mediating role of social support.

**Methods:**

We used the 3-waves of COVID-19 survey within the Millennium Cohort Study (MCS, age 19, *N* = 5522) and MCS Parent (MCSP, age > > 19, *N* = 7479) samples, with additional pre-pandemic measures of some outcomes and exposure. Using Structural Equation Panel Models with Full Information Maximum Likelihood estimation to address missing data, we studied differences between respondents with a chronic illness and without, regarding depressive symptoms and mental well-being, with social provision, social support, and loneliness as potential mediators.

**Results:**

Mental well-being (SWEMWBS) and psychological distress (Kessler-6) worsened significantly during the pandemic relative to baseline for people with and without chronic illness, while the latter group had substantially better well-being at all waves and the baseline regarding both outcomes. When the lockdown was lifted during wave-2, mental well-being temporarily rebounded, and distress waned among people without chronic illness but continued to worsen among people with chronic illness. Social support partially mediated the link between chronic illness and mental well-being.

**Conclusions:**

The large mental well-being gap between people with and without chronic illness persisted during the pandemic. However, social support and provision can partially narrow this gap, hence should be employed in future pandemic management.

**Supplementary Information:**

The online version contains supplementary material available at 10.1186/s40359-024-01596-x.

## Introduction

The COVID-19 pandemic and public health measures to contain it, such as distancing, lockdowns, and quarantines, are thought to have reduced the population’s social capital, while social capital acts as an important buffer of stress [1–3]. The pandemic and related measures have been associated with increases in stress, anxiety and depression due to, for example loneliness and reduced social support, worsening economic prospects, and future unpredictability [4–7].

Most research has focused on consequences for the general population. Less attention has been paid to people with pre-existing chronic mental and physical illnesses [8]. Their long-standing chronic conditions, without immediate cure, will in many cases have affected their daily living [9], and thus they would have likely experienced the pandemic differently compared with the general population. Indeed, the inequitable impact on this group was acknowledged early in the pandemic [10, 11]. The scarcity of research concerning the pandemic impact on people with chronic illness, which we summarise next, is thus surprising.

A few studies have noted possible pandemic resilience among people with some chronic conditions [12–16]. Certain developments during the pandemic may also have helped some people with chronic conditions. These include increased use of online support and activities [17], better guidance for and diffusion of self-management tools for tasks previously done by care services [18], and in some cases, improved adherence to medications [19, 20]. However, reports suggest these positive impacts to be relatively rare [21, 22].

In early 2020, approximately 16% of people in the UK had at least one mental or physical chronic health condition [23]. Those who were previously well-supported by healthcare providers, family, and friends, have seen a reduction in this support during the pandemic, while existing challenges have worsened for others. There has been mostly poor treatment adherence [24–27] and lack of self-care, exacerbated by concerns about access to remote health care and a reduction in social trust [28–30]. There has also been an increased risk of isolation [31, 32, 33] and abuse or neglect [34]. Pre-existing psychosocial factors have led to a reduced capacity in this group to cope with social, economic and psychological pandemic impacts compared with the population overall [33]. People with chronic conditions may also be particularly vulnerable economically, often unemployed or on low-wage or zero-hour contracts [20].

Given the importance of considering the pandemic impacts on people with chronic conditions and the relative dearth of studies that focus on these, we have been undertaking a longitudinal study in the UK to fill some of the gaps in the evidence base. Our aim is to develop an in-depth understanding of the pandemic-relevant mental and physical health, social and health care support, and other experiences of people with chronic conditions/disabilities. In the present article, which is part of this wider study, we report on a secondary analysis of data from the longitudinal UCL COVID-19 survey.

We aim to contribute to the literature on three fronts. Firstly, focusing on people with chronic conditions and comparing their mental health and social capital with those who do not have chronic conditions, we address the aforementioned gap. We hypothesise that people with chronic conditions will report higher levels of anxiety and psychological stress, loneliness, and lower levels of social support during the COVID-19 pandemic than those without these conditions. We base this expectation on the results from prior studies that primarily targeted the general population. For example, prior mental and physical health diagnoses have been found to be associated with lower levels of well-being and loneliness [35]. Levels of social support have been shown to be relatively constant through the pandemic but reduced during periods of restrictions and certain groups of people (e.g. living alone, poorer, less educated, from minoritised ethnic groups, and with a pre-existing mental or physical health diagnosis) have consistently experienced lower levels of support [36].

Secondly, we conduct a mediation analysis to understand whether and how social support, loneliness, and social provision play a role in mental health differences between people with and without chronic conditions. Social support has been reported to not only improve well-being before and during the pandemic [37, 38] but also to mediate the relationship between loneliness, pre-existing health conditions and poor mental health [39–42]. This mediation analysis will help develop strategies to mitigate the inequitable impact the pandemic may have had on people with chronic conditions, during the post-pandemic recovery phase.

Thirdly, we rely on good quality longitudinal data, which are collected as an extension of an existing large-scale cohort study. This offers two key methodological advantages and significant improvements over most existing panel studies conducted during the pandemic. Firstly, our data allow us to not only adjust our statistical models with the baseline measures of some of the outcomes but also gauge possible bias due to differential attrition or self-selection in the pandemic waves of the panel. Secondly, the data cover a large socio-economic and geographical landscape, nationally representing the analysed cohort.

## Methods

### Study design and participants

The UCL COVID-19 survey has been conducted with participants across five cohort studies maintained by University College London. Not all variables were measured in all cohorts. Here we focus on the survey conducted with the Millennium Cohort Study (MCS) participants and their parents (MCSP) for the availability of variables of interest. MCS has been following the lives of around 19,000 people born in the UK in 2000-02 and of their parents. The UCL COVID-19 survey has been administered to the MCS and MCSP respondents thrice. Waves 1, 2, and 3 took place respectively in May 2020, September-October 2020, and February-March 2021. Hence, wave 1 was implemented during the initial UK lockdown, while wave 2 took place when restrictions were eased. Wave 3 happened during the third lockdown. Waves 1 and 2 took place entirely online while a minority of wave 3 respondents (20% MCS and none of the MCSP) were interviewed via telephone; the rest were interviewed online. No links were made between the MCS respondents and their parents (MCSP) during invitation or within the questionnaire. For further details of the UCL COVID-19 survey, see [43, 44] and for a recent example [45]. Supplementary Information (SI) 1 in the online appendix shows sample characteristics.

We merged the three waves of the MCS and MCSP COVID-19 surveys with the most recent pre-pandemic wave, which happened in 2018 when the MCS participants were aged 17 (henceforth wave 0).

### Measures

#### Outcomes

Mental well-being is measured by the Shortened Warwick-Edinburgh Mental Well-being Scale (SWEMWBS). This is a unidimensional construct capturing positive well-being [46, 47]. It comprises seven questions about feelings and thoughts in the past two weeks (e.g. “I’ve been feeling useful”). Responses are given on a 5-point Likert scale from “none of the time” to “all the time”. Cronbach’s α in both MCS and MCSP is over 0.80 in all waves. For our analysis, we used a SWEMWBS mean score, which is between 1 and 5, with higher scores indicating better well-being. SWEMWBS is included in all waves and the baseline in MCS, and in all waves but the baseline in MCSP.

Psychological distress is measured with the Kessler-6 scale [48], comprising six questions about symptoms the respondent experienced in the last 30 days (e.g. “how often did you feel hopeless?”) Answer categories range from 1 “none of the time” to 5 “all of the time”. Cronbach’s α for both MCS and MCSP is consistently close to 0.9. We created a Kessler-6 mean score (range 1–5), higher scores indicating more frequent distress. Kessler-6 is available for both MCS and MCSP in all waves and the baseline.

#### Exposure

Chronic illness is measured with the question “do you have any of the following?” answers listing 16 of common long-standing conditions. These are: (1) Cancer; (2) Cystic fibrosis; (3) Asthma; (4) Chronic Obstructive Pulmonary Disease; (5) Wheezy bronchitis; (6) Diabetes; (7) Recurrent backache, prolapsed disc, sciatica or other back problem; (8) Problems with hearing; (9) High blood pressure; (10) Heart disease, congenital or acquired; 11) Depression or other emotional, nervous or psychiatric problems; 12) Obesity; 13) Chronic obstructive airways disease; 14) Infection; 15) HIV / Immunodeficiency, and 16) Condition affecting the brain and nerves. SI2 shows the number of people by sample and wave who have each of these 16 conditions. A binary item indicates whether the respondent has any of the listed 16 long-standing illnesses or not. In additional analyses, we further differentiate “psychological” chronic illness (condition 11) and “physical” chronic illness (any others in the list). Exposure is measured in waves 0, 1, 2, 3 for MCS and 1, 2, 3 for MCSP.

#### Mediators

The Short Social Provisions Scale measures social support through 3 items how much the respondent thinks “[they] have family and friends who help [them] feel, safe, secure and happy”, “there is someone [they] trust whom [they] would turn to for advice if… having problems”, and “there is no one [they] feel close to” [49]. Answers are from 1 “very true” to 3 “not at all true”. Cronbach’s α is 0.7 across the two samples and three waves. We created a reverse coded average score (after reverse coding item-3), with higher scores indicating more support. This scale is available for waves 0, 1, 2, 3 for MCS, and 1, 2, 3 for MCSP.

Specific social support is measured with the question “if you were sick in bed, how much could you count on the people around you to help out?” with 4-category Likert answers from 1 “not at all” to 4 “a great deal”. This measure is available for both MCS and MCSP in all waves but the baseline.

Finally, the UCLA Loneliness Scale (3-items) is used to measure loneliness and social isolation [50]. The items comprise “How often do you feel that you lack companionship?”, “How often do you feel left out?”, and “How often do you feel isolated from others?”. Responses are from 1 “hardly ever” to 3 “often”. Mean scores range from 1 to 3, higher scores indicating stronger feelings of loneliness and social isolation (Cronbach’s α is consistently over 0.80 across the samples and waves). This measure likewise is available for both MCS and MCSP in all waves but the baseline. SI3 shows the descriptive statistics of the outcome and mediators by sample and wave.

#### Controls

Several important factors may have affected mental wellbeing during the pandemic. Moreover, as the pandemic evolved, respondents’ attitudes toward risk or actual risks they face may have contributed to their wellbeing. If these factors confound the links between the exposure and the outcome and between the mediators and the outcome, not accounting for them may result in bias. Note however that our dataset is longitudinal, and we allow for correlations between the error terms of all endogenous variables as explained below. These correlations should account for such confounding to some extent. Nevertheless, in our final analysis, we adjust for the following five factors: whether the respondent reported having had Covid by each wave, whether any close relative or friend of the respondent died post-pandemic (available for wave 2 and 3), self-assessed willingness to take risks (0 = never to 10 = always) by wave, whether the respondent received a shield letter identifying them as at risk by each wave, and likelihood of choosing to be vaccinated if offered (0 = not at all, 4 = very). The last variable is measured only in wave 3.

### Analysis

The number of respondents varies across the three waves due to attrition or new respondents joining in (see SI3). To address the possible impact of missing data on bias and statistical power, we use Full Information Maximum Likelihood (FIML) estimation in all our analyses. FIML is shown to produce unbiased estimates when data are missing at random (i.e. missingness depends only on the observed data) and follow a multivariate normal distribution. Research shows that FIML is rather robust to violations of multivariate normality [51].

**Fig. 1:**
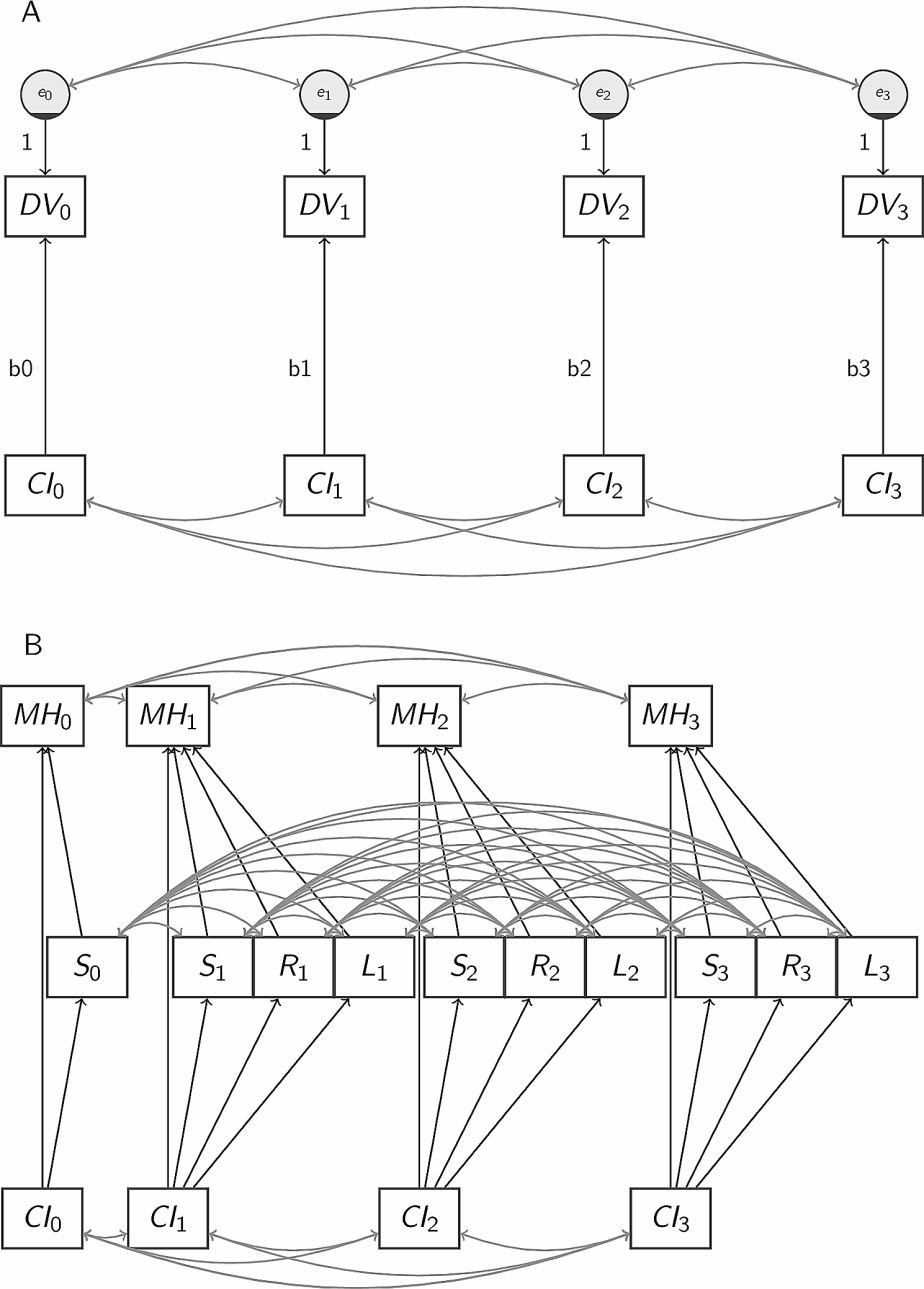
Panel Data Structural Equation Models. DV represents a generic outcome (Dependent) Variable; CI = participant has Chronic Illness, MH = Mental Health (SWEMWBS or KESSLER), S = Social provision score, R = specific social support (can Rely on someone when sick), L = Loneliness. Subscripts indicate the survey wave: 0 is the pre-pandemic wave, wave 1 is the first lockdown, wave 2 is between lockdowns, wave 3 is the third lockdown. Panel A shows the model used to estimate the trends in outcomes and mediators across the waves. Panel B shows the longitudinal mediation model (residual terms associated with endogenous variables are suppressed for brevity). For certain variables, a baseline measure (wave 0) is missing, in which case the model excludes the baseline variable and all paths associated with it

Modelling is implemented in two steps. First, we fitted the model shown in Fig. 1-A that estimates the association between chronic illness and a generic outcome variable (i.e. SWEMWBS or Kessler-6), whereby the path from chronic illness to the outcome is allowed to vary across the three waves. The model in Fig. 1-A also allows correlations between the residuals of the outcome and of the exposure across the three waves, capturing unaccounted common causes of the outcome and of the exposure. This model is used to estimate the trends in the two outcome variables (SWEMWBS or Kessler-6) and the three mediators (social provision, social support and loneliness). When a variable lacks a baseline measure (wave 0), it and associated paths are excluded from the model. Also, chronic illness is not measured for the MCSP at the baseline, while Kessler-6 is. In that case, the baseline outcome is regressed on the exposure in wave 1.

Then, we fitted the mediation model shown in Fig. 1-B, whereby mental health (measured either by SWEMWBS or Kessler-6) is regressed on chronic illness and the mediators (in wave 0, only one mediator because of data availability). The model allows correlations between different measurement occasions of the same variable for all variables, and the correlations between all mediators across all waves, as shown in Fig. 1-B. The model is slightly modified for MCSP. First, SWEMWBS is missing for MCSP at wave 0, so a reduced form of Fig. 1-B is fitted for MCSP. Second, the baseline Kessler-6 value is regressed on the exposure in wave 1 (instead of wave 0), and the mediator in wave 0 is removed due to data availability.

A comparison of the paths from chronic illness to SWEMWBS and those to Kessler-6 between the model in Fig. 1-A and in Fig. 1-B will indicate how much of the total effect estimated in the former is accounted for by the mediators in the latter.

Finally, as an additional analysis we distinguish psychological and physical chronic illness and expand on the models in Fig. <link rid="fig1”>1</link>-A and 1-B with the two binary indicators of exposure (psychological and physical). In the mediation version of these additional analyses, we also collapse, for simplicity, MCS and MCSP samples while still controlling for the sample. We then also add control variables in this analysis whereby all outcome variables and the mediators are regressed on these controls.

### Mediation versus moderation

As discussed in the introduction, we expect that people with chronic conditions will report worse well-being (SWEMWBS) and higher psychological distress (Kessler-6) throughout the pandemic (H1 and H2, respectively). Moreover, based on prior studies that primarily targeted the general population which showed that social support was lower among certain, mostly disadvantaged groups [35, 36], we expect lower levels of social provision (H3) and specific social support (H4) and higher levels of loneliness (H5) among people with chronic conditions than without. Because social support is shown to improve well-being before and during the pandemic [37, 38] and also to mediate the relationship between pre-existing disadvantages [39–42], we expect the negative effects of chronic conditions on wellbeing and the positive effects on distress predicted in H1 and H2 to be, at least partially, mediated by the reduction in support and increase in loneliness predicted in H3, H4, and H5. Overall, thus, we hypothesize the mediation model shown in Fig. 1-B. Note that the mediation model in Fig. 1-B implies that reduced social provision and, specific social support, and increased loneliness will have detrimental effects on well-being and distress (respectively H7, H8, H9).

One could argue for an alternative theoretical model whereby social support and loneliness do not *mediate* the link between chronic illness and well-being but *moderate* it. A moderation model would imply *interaction effects*, that is the effects of support and loneliness on well-being and distress would vary between people with and without chronic illness. Yet, we hypothesise that social support is beneficial, and loneliness is detrimental for everyone to a comparable degree, irrespective of chronic illness status, but that people with chronic illnesses had lower levels of support and higher levels of loneliness. Hence, our model specifies mediation rather than moderation. We nevertheless present formal tests of moderation by specifying appropriate interaction effects.

## Results

**Fig. 2:**
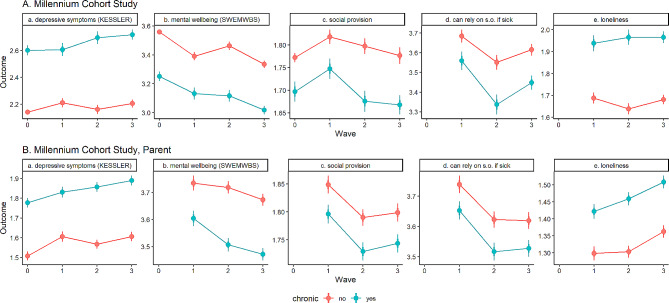
Estimated means of the outcome and mediating variables (+ 95% CI & SE) among people with and without chronic illness in the four waves for the MCS and MCS Parent samples. Wave 0 is pre-pandemic, wave 1 is the first lockdown, wave 2 is between lockdowns, wave 3 is the third lockdown. Mean estimates and their standard errors are obtained with the panel model given in Fig. 1-A

Figure 2 shows the trends in the outcomes (SWEMWBS and Kessler-6) and the mediators (social provision, specific social support, and loneliness) among people with and without chronic illness in the three waves and baseline whenever available. All differences between people with and without chronic illness in all outcomes and mediators in all waves are statistically significant. People with chronic illness have significantly more frequent distress (H2), worse mental well-being (H1), poorer social support (H3, H4), and stronger feelings of loneliness (H5) in all waves compared with people without any chronic illness.

While generally, mental well-being and psychological distress worsen during the pandemic for both people with and without chronic illness, time trends are nonlinear and different between the two groups. People with and without chronic illness diverge regarding SWEMWBS and Kessler-6 scores, most notably in wave 2, during the relative relaxation of pandemic restrictions. In wave 2, compared with wave 1 which happens during the 1st lockdown, people without chronic illness have improved SWEMWBS in MCS [difference is 0.07, 95% CI: (0.04, 0.11)] and lower Kessler-6 scores both in MCS [difference = − 0.05, 95% CI: (-0.09, − 0.01)] and in MCSP [difference = − 0.04, 95% CI: (-0.08, − 0.00)]. However, in the same period, among people with chronic illness, mental well-being worsened in MCSP: the difference between wave 2 and wave 1 in SWEMWBS = − 0.10, (95% CI: − 0.13, − 0.07) and psychological distress worsened in MCS: the difference between wave 2 and wave 1 in Kessler-6 score is 0.09 (95% CI: 0.03, 0.16).

Table 1 presents a selection of the estimated path coefficients in the model in Fig. 1-B (for the full set of results see SI4 and SI5). For comparison, the table also present corresponding path coefficients estimated using the model in Fig. 1-A when the outcomes are SWEMWBS and Kessler-6 —these path coefficients represent the total effect of chronic illness on health and well-being. Table 1 also includes selected fit measures that indicate reasonably good model fit. While Chi-squares are statistically significant, this is expected given the relatively large samples. RMSEA and, to some extent, CFI indicate acceptable fit.

**Table 1:** Estimated path coefficients and fit measures for models in Fig. 1 where the outcome variable is mental well-being (SWEMWBS) and psychological distress (KESSLER). Only path coefficients for Wave 2 variables are shown for brevity, other path coefficients are similar (see SI4 and SI5 for the full set of parameters). Fitted with Full Information Maximum Likelihood. **p < 0.01. The hypothesis with the predicted sign is given in parentheses next to the predictor variable

	**Outcome: mental well-being**											
	MCS Sample						MCS Parent Sample					
	M1			M2			M3			M4		
SWEMWBS 2 on	b	se		b	se		b	se		b		se
Chronic illness 2 (H1−)	− 0.345**	0.025		− 0.171**	0.022		− 0.212**	0.017		− 0.117**		0.014
Can rely on so 2 (H8+)				0.039**	0.007					0.079**		0.007
Social provision 2 (H7+)				0.461**	0.015					0.219**		0.014
Loneliness 2 (H9−)				− 0.423**	0.011					− 0.502**		0.011
Can rely on so 2 on												
Chronic illness 2 (H4−)				− 0.146**	0.031					− 0.075**		0.020
Social provision 2 on												
Chronic illness 2 (H3−)				− 0.102**	0.014					− 0.051**		0.011
Loneliness 2 on												
Chronic illness 2 (H5+)				0.293**	0.021					0.147**		0.013
N	5552			5552			7479			7479		
Chi2(df)	273.393(12)			987.854 (79)			81.726 (6)			420.358 (48)		
CFI	0.961			0.959			0.990			0.985		
RMSEA (90% CI)	0.062 (0.056-0.069)			0.043 (0.041-0.046)			0.041 (0.033-0.049)			0.032 (0.029-0.035)		
				**Outcome: Psychological Distress**								
	MCS Sample						MCS Parent Sample					
	M5			M6			M7		M8			
KESSLER 2 on	b		se	b		se	b	se	b		se	
Chronic illness 2 (H2+)	0.532**		0.029	0.357**		0.026	0.289**	0.016	0.224**		0.015	
Can rely on so 2 (H8−)				− 0.003		0.009			− 0.045**		0.007	
Social provision 2 (H7−)				− 0.469**		0.018			− 0.082**		0.014	
Loneliness 2 (H9+)				0.555**		0.013			0.470**		0.011	
Can rely on 2 on												
Chronic illness 2 (H4−)				− 0.139**		0.031			− 0.072**		0.019	
Social provision 2 on												
Chronic illness 2 (H3−)				− 0.094**		0.014			− 0.051**		0.011	
Loneliness 2 on												
Chronic illness 2 (H5+)				0.277**		0.021			0.143**		0.013	
N	5552			5552			7496		7496			
Chi2(df)	515.763 (12)			1586.850 (79)			166.285 (8)		1670.899 (59)			
CFI	0.942			0.932			0.986		0.944			
RMSEA	0.087 (0.081-0.092)			0.059 (0.056-0.062)			0.051 (0.045-0.058)		0.060 (0.058-0.063)			

The mediation results in Table 1 show that social provision, specific social support, and loneliness and social isolation partially mediate the link between chronic illness and well-being and that between chronic illness and psychological distress. The paths from chronic illness to SWEMWBS in all waves are effectively halved in M2 (mediation model) compared to M1 (association between chronic illness and the outcome) for MCS sample and in M4 (mediation model) compared to M3 (association between chronic illness and the outcome) for MCSP sample (with an exception in wave 0 for which we only have a single mediator in MCS). The paths from chronic illness to Kessler-6 in all waves also decrease but not as strongly as they do for SWEMWBS when mediators are considered, again for both MCS and MCSP. The direct paths from chronic illness to SWEMWBS and Kessler-6 remain strong and statistically significant for all waves after including mediators in the model. These results show partial mediation.

**Fig. 3:**
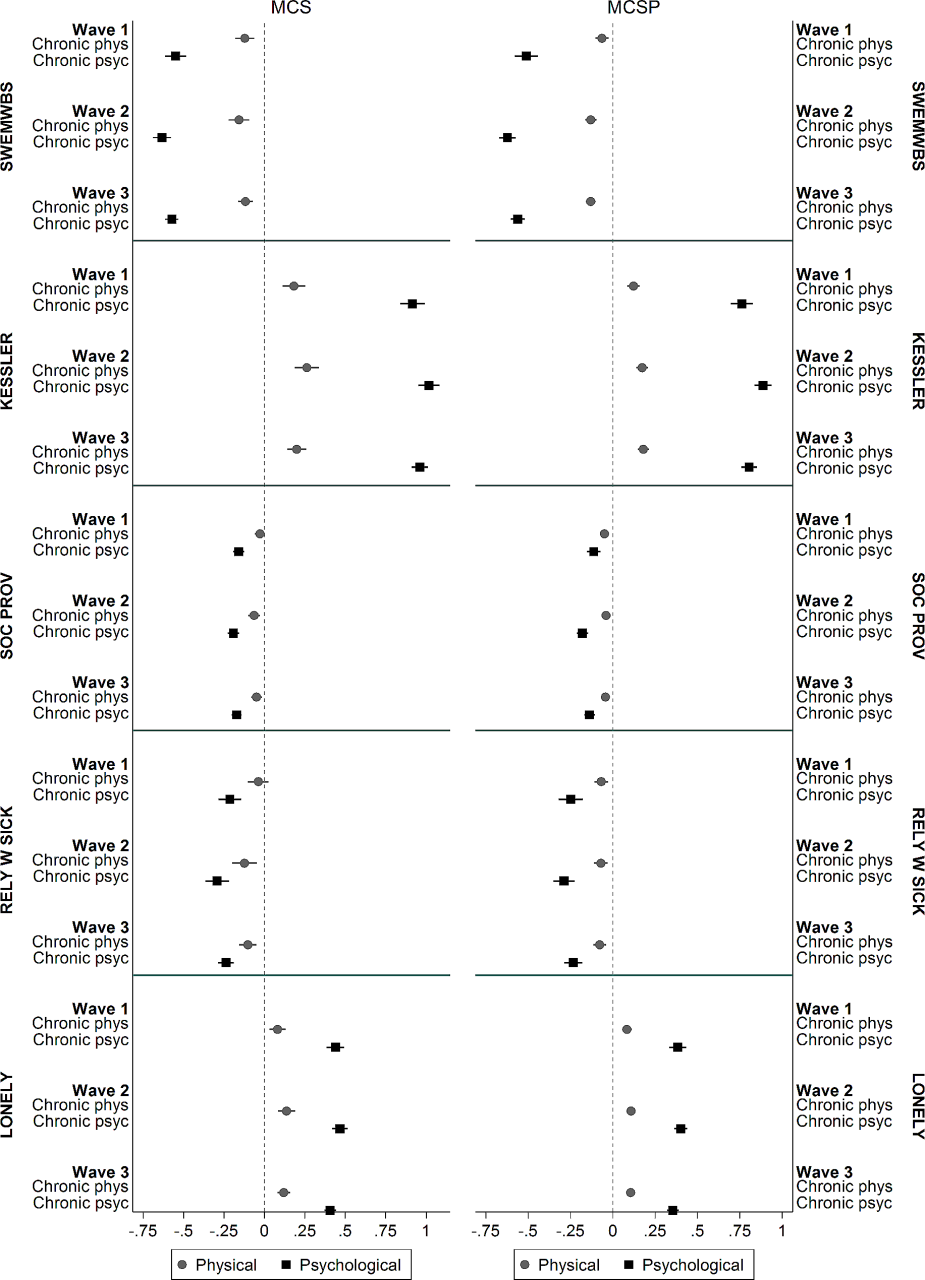
Differences between people with and without chronic conditions by survey wave (+ 95% CI) broken down by condition type: physical or psychological and by sample (MSC and MSCP). Estimates are obtained with the panel model given in Fig. 1-A, after removing wave zero

Finally, our outcomes are mental well-being and psychological distress while one of the categories of chronic illness also include depression or other emotional, nervous or psychiatric problems. To assess how people with other forms of chronic illness fared vis-à-vis people without any chronic condition, we distinguish psychological and physical chronic illness. Figure 3 shows the differences between people with and without chronic conditions in the three COVID-19 waves and sample (MCS and MCSP), broken down by condition type: physical or psychological (note that some people may have both forms, hence these are not mutually exclusive groups). These differences are estimated using an expanded model in Fig. 1-A. Figure 3 shows that, unsurprisingly psychological chronic illness is much more detrimental than other physical forms of chronic illnesses. However, physical chronic conditions too are detrimental, for people with such conditions have in nearly all waves significantly worse well-being, distress, and feelings of loneliness, and lower social provision and support than people without any chronic conditions. In addition, the trends for people with physical chronic illness in the three COVID-19 waves are very much parallel to those with psychological chronic illness. Hence, while “levels” of well-being and psychological distress for people with psychological and physical chronic illnesses differ, changes during the pandemic do not seem to differ.

Table 2 likewise shows a selected set of parameters (full results are in SI6) for the mediation model which distinguishes psychological and physical chronic illness. The results again confirm that both psychological and physical chronic illness have been detrimental regarding wellbeing and distress during the pandemic, while the former form is more so. Moreover, social provision, support, and loneliness mediate the effects of both psychological and physical chronic conditions on well-being and distress (for both forms of chronic illness, coefficients reduce nearly by half after including the mediators, compare M1 with M2 and M4 with M5 in Table 2). Adjusting for the five control variables hardly affects the existing coefficients (M3 and M6), suggesting that the results are robust to confounding.

**Table 2:** Estimated path coefficients for models in Fig. 1 fitted to the merged MCS and MCSP samples where the outcome variables are mental well-being (SWEMWBS) and psychological distress (KESSLER); and the main independent variable chronic illness has two forms, psychological and physical; only parameters for Wave 2 are shown for brevity, other path coefficients are similar (see SI6 for full set of parameters). Fitted with Full Information Maximum Likelihood. **p < 0.01, +*p* < 0.1. See Table 1 for hypotheses and predicted coefficient sign

Outcome:	SWEMWBS						KESSLER					
	M1		M2		M3		M4		M5		M6	
SWEMWBS /KESSLER on	b	se	b	se	b	se	b	se	b	se	b	se
Can rely on so			0.066**	0.005	0.063**	0.005			− 0.041**	0.006	− 0.038**	0.006
Social provision			0.258**	0.011	0.255**	0.011			− 0.153**	0.012	− 0.149**	0.012
Loneliness			− 0.477**	0.008	− 0.460**	0.008			0.605**	0.009	0.594**	0.009
MCS (vs. MCSP)	− 0.301**	0.011	− 0.131**	0.009	− 0.153**	0.009	0.613**	0.012	0.398**	0.010	0.417**	0.011
Chronic illness (psychological)	− 0.599**	0.019	− 0.335**	0.017	− 0.314**	0.017	0.954**	0.020	0.666**	0.018	0.665**	0.018
Chronic illness (physical)	− 0.098**	0.015	− 0.054**	0.010	− 0.043**	0.013	0.116**	0.016	0.070**	0.014	0.070**	0.013
Had covid					− 0.015	0.016					0.037**	0.011
Relative died since pandemic					− 0.036*	0.015					0.040**	0.011
Vaccine acceptance					0.012	0.008					− 0.010	0.006
Risk attitude					0.050**	0.003					− 0.030**	0.002
Shield for covid risk					− 0.027	0.030					0.061**	0.021
Can rely on so on												
MCS (vs. MCSP)			− 0.050**	0.011	− 0.027*	0.012			− 0.048**	0.012	− 0.025*	0.012
Chronic illness (psychological)			− 0.301**	0.024	− 0.278**	0.024			− 0.293**	0.023	− 0.279**	0.024
Chronic illness (physical)			− 0.046**	0.013	− 0.040*	0.017			− 0.032+	0.016	− 0.023	0.017
Had covid					− 0.003	0.024					− 0.003	0.014
Relative died since pandemic					0.013	0.023					− 0.018	0.014
Vaccine acceptance					0.106*	0.011					0.090**	0.007
Risk attitude					0.024**	0.004					0.017**	0.002
Shield for covid risk					− 0.085*	0.042					− 0.026*	0.011
Social provision on												
MCS (vs. MCSP)			− 0.002	0.006	0.009	0.007			0.000	0.007	0.010	0.007
Chronic illness (psychological)			− 0.187**	0.012	-0.172**	0.012			− 0.191**	0.012	− 0.179**	0.012
Chronic illness (physical)			− 0.037**	0.008	-0.032**	0.008			− 0.030**	0.008	− 0.027**	0.009
Had covid					0.007	0.012					0.018*	0.008
Relative died since pandemic					− 0.003	0.012					− 0.003	0.007
Vaccine acceptance					0.059**	0.006					0.047**	0.004
Risk attitude					0.014**	0.002					0.011**	0.001
Shield for covid risk					− 0.060**	0.021					− 0.017**	0.006
Loneliness on												
MCS (vs. MCSP)			0.438**	0.016	0.358**	0.009			0.360**	0.009	0.361**	0.009
Chronic illness (psychological)			0.356**	0.008	0.413**	0.015			0.441**	0.015	0.403**	0.016
Chronic illness (physical)			0.077**	0.010	0.066**	0.011			0.099**	0.010	0.079**	0.010
Had covid					− 0.004	0.016					− 0.009	0.010
Relative died since pandemic					0.014	0.015					0.021*	0.009
Vaccine acceptance					− 0.050**	0.007					− 0.040**	0.005
Risk attitude					− 0.032**	0.002					− 0.028**	0.002
Shield for covid risk					0.111**	0.027					0.067**	0.022

### Additional analyses

COVID-19 surveys had to be implemented quickly and often online with convenience samples [52, 53]. Given that people with underlying risk factors and different mental well-being levels can opt into such surveys with different propensities, existing results reported in the literature potentially suffer from sample selection bias. Importantly, because the COVID-19 survey we use is implemented on an existing cohort study, we can test potential selection bias.

Of the nearly 24,000 respondents (MCS and MCSP combined) who participated in the 2018 wave (wave 0), 13,000 participated in at least one of the later Covid-9 survey waves. We tested whether SWEMWBS and Kessler-6 scores in wave 0 (baseline) varied among people who participated in any of the later COVID-19 surveys, and more importantly, whether any difference differed between people with and without chronic illness (i.e. interaction between chronic illness and non-attrition). While we find significant differences at the baseline between people who appeared in the later COVID-19 surveys (interestingly, people who participated in the later COVID-19 surveys had more frequent depressive symptoms and lower SWEMWBS at the baseline than people who did not participate further), the interaction between attrition and chronic illness is statistically highly insignificant [F(1,9844) = 0.07, *P* = 0.784 for Kessler-6 at wave 0; F(1,9840) = 0.17, *P* = 0.683 for SWEMWBS at wave 0]. We thus conclude that potential sample selection issues do not bias the association between chronic illness and well-being here.

We finally test moderation by adding interaction effects between the indicator for chronic illness on the one hand and on the other social provision, specific social support, and loneliness in the models that predict SWEMWBS and Kessler-6 in wave 3. We also adjust for the baseline outcome and address missing data with FIML. We do not find enough evidence in our data for moderation, neither for SWEMWBS [χ^2^ (3)  = 7.35, *P* = 0.062 for MCS and χ^2^ (3)  = 1.69, *P* = 0.640 for MCSP] nor for Kessler-6 [χ^2^ (3)  = 1.14, *P* = 0.769 for MCS and χ^2^ (3)  = 6.24, *P* = 0.101 for MCSP].

## Discussion

Despite the increased burden of the pandemic on people with pre-existing mental and physical chronic health conditions having been acknowledged from early 2020 [10, 11], subsequent studies have not focused in any depth on this group. Here we attempted to address this gap by focusing on people with chronic conditions and comparing their mental well-being and social capital during and before the pandemic with those who do not have chronic conditions. Additionally, we conducted a mediation analysis to study how differences in social support, loneliness, and social provision may explain the mental well-being gap between people with and without chronic conditions. We did so using good quality nationally representative longitudinal data, which included pre-pandemic measures of some key variables. The data we used, thus, offered important improvements over the existing large-scale panel studies conducted during the pandemic, which relied on convenient online samples or lacked pre-pandemic baseline measures.

We found that depressive symptoms and mental well-being significantly worsened during the pandemic, relative to baseline for people with and without a chronic condition. Those without a chronic condition had substantially better well-being at baseline and all subsequent waves compared to people with chronic conditions. When the lockdown was lifted during wave 2, mental well-being temporarily rebounded, and depressive symptoms eased among people without chronic conditions but continued to worsen for people with chronic conditions. Thus, the gap between those with and without chronic illness regarding well-being, psychological distress, access to social support and provision, and loneliness increased throughout the pandemic. Social support and provision, and loneliness partially mediated the link between chronic illness and mental well-being and health.

Overall, our results on trajectories of depression (e.g. 53, 35) and the mediating role of social support and provision correspond (e.g. [39–42]) with those found in the literature.

However, there were some differences in other outcomes of interest. Past research reported generally stable levels of loneliness during the pandemic [52]. However, we found a trajectory of constant worsening of mental well-being and loneliness for the parent cohort (MCSP) irrespective of whether respondents had chronic conditions or not, while for the young adults (MCS) only when they had a chronic condition. The COVID-19 Social Study by [35], which is used by [52], covered a slightly longer period than our data, but both sets of data cover broadly similar pandemic episodes. The COVID-19 Social Study had a mean age of 49 years, roughly equivalent to our MCSP. However, it may be relevant that the adults in the COVID-19 Social Study were self-selected from the overall population, whereas the MCSP survey considered parents specifically. Therefore, MCSP respondents have been parents of children, and some of these children have had chronic conditions. Considering that some parents may also have had a chronic condition themselves (especially if it was hereditary), more MCSP respondents may have been carers or both sufferers and carers. Indeed a few studies showed that a constant decline in well-being was associated with being a carer amongst other factors [54] and that carers experienced stronger deterioration of mental health than non-carers [55]. Irrespective of their causes, however, these results show the complexity of the situation and the importance of focusing on people with chronic conditions and their carers. Recovery seems absent in these groups; thus, intervention is needed to prevent widening health inequalities.

### Limitations

While we can separate psychological and physical chronic conditions, lack of distinction between illness severity and limiting chronic conditions to 16 common ones in the data might obscure those that do not fit in the list of 16. Different chronic illnesses and severities would demand different needs [56, 57], which may shape the pandemic experience. Our priority is to document differences between people with chronic illness of psychological and physical type and those without during the pandemic. Future analyses will need to disentangle further different types of chronic illnesses and their specific impacts during the pandemic on both those with chronic conditions and their carers.

Second, the data do not allow for distinguishing different types of social provision (e.g., online versus face-to-face). Sommerlad et al. [35] found that while a range of social provision types was protective against depression during the pandemic, the effects varied across the types. Moreover, the reliability of the social provision scale is acceptable (α = 0.7) but lower than other measures. Hence, its coefficients could be biased downward due to measurement error.

Third, we consider two specific age groups, young people born in 2000 and their parents who are likely to represent a limited age range and whose children are likely in many cases to be transitioning to independent or semi-independent living. Therefore, both age groups are at a transition point in their lives which affect mental wellbeing [58, 59]. Hence, extending our worth to other age groups will be useful. Additionally, while waves 1 and 2 took place entirely online, a minority of wave 3 data comes from telephone interviews (20% MCS and none in MCSP). While this shift in mode presents a limitation, it stems from the challenges the pandemic posed for rapid data collection.

### Clinical and research implications

Our findings highlight the significant and persistent differences between people with chronic condition and those without in social support, loneliness, social provision, and mental health during the COVID-19 pandemic, calling for an urgent need for improving the conditions of people with chronic conditions. People with chronic conditions are more susceptible to experiencing mental distress than the general population, even pre-pandemic [60]. Interventions and preventive initiatives targeting this group are thus crucial. Our results reveal the mediating role of social support and isolation in the chronic illness-mental well-being and health link. Interventions can thus focus on social support to increase resilience among individuals with chronic illness. Such interventions could be, in the short run, providing support and training in developing skills in self-management, advocacy for needs, seeking practical support from the community, and building networks and problem-solving with professionals. In the mid run, raising awareness among health practitioners for implementing better interaction plans and allowing flexibility in mode (face-to-face or online) will be helpful. Likewise, mobilising social prescribing and improving community centre offerings are important. Finally, in the long run, creating a larger safety net for people with chronic illness will allow them to seek better support and services.

### Electronic supplementary material

Below is the link to the electronic supplementary material.

Supplementary Material 1

## Data Availability

All data used in this study are publicly available at the UK Data Service (https://beta.ukdataservice.ac.uk/datacatalogue/studies/study?id=8658). Access to the database is open.
