# Supplementary material for: Social support and mental well-being among people with and without chronic illness during the Covid-19 pandemic: evidence from the longitudinal UCL covid survey
Source: BMC Psychol. 2024 Mar 11;12:136. doi: 10.1186/s40359-024-01596-x (PMC10929080; doi:10.1186/s40359-024-01596-x)
Supplement: Supplementary file 1 — Supplementary Material 1 [file 40359_2024_1596_MOESM1_ESM.docx]

# **Supplementary Information for “Social support and mental well-being among people with and without chronic illness during the Covid-19 pandemic: Evidence from the longitudinal UCL covid survey”**

Contents

[Supplementary Information for “Social support and mental well-being among people with and without chronic illness during the Covid-19 pandemic: Evidence from the longitudinal UCL covid survey” 1](#_Toc106908433)

[SI1: Sample characteristics 2](#_Toc106908434)

[SI2: Number and proportion of respondents with each chronic illness 5](#_Toc106908435)

[SI3: Descriptive statistics of the MCS and MCSP samples broken down by survey wave 6](#_Toc106908436)

[SI4: Full set of path coefficients for models in Figure 1: outcome SWEMWBS 7](#_Toc106908437)

[SI5: Full set of path coefficients for models in Figure 1: outcome KESSLER 8](#_Toc106908438)

[SI6: Full set of parameters of models given in Table 2 in main manuscript 9](#_Toc106908439)

## SI1: Sample characteristics

|  |  | **MCS Parents** | | | **MCS Cohort Members** | | |
| --- | --- | --- | --- | --- | --- | --- | --- |
|  |  | **W1** | **W2** | **W3** | **W1** | **W2** | **W3** |
| **Age** | **Mean(SD)** | 54.2(5.4) | 54.1 (5.8) | 54.2 (5.7) | - | - | - |
| **Gender** | **Male%** | 893(31.6%) | 1848 (34.8%) | 1834 (35.0%) | 775 (29.4%) | 1050 (35.0%) | 1755 (39.3%) |
|  | **Female** | 1936 (68.4%) | 3467 (65.2%) | 3412 (65.0%) | 1865 (70.6%) | 1951 (65.0%) | 2709 (60.7%) |
| **Ethnicity N(%)** | **White** | 2644(95%) | 4606 (92.8%) | 4326 (93.7%) | 2195 (86.5%) | 2723 (86.1%) | 3627 (84.0%) |
|  | **Black African** | 20(0.7%) | 49 ( 1.0%) | 40 ( 0.9%) | 37 ( 1.5%) | 49 ( 1.5%) | 74 ( 1.7%) |
|  | **Black Caribbean** | 18(0.6%) | 25 ( 0.5%) | 24 ( 0.5%) | 21 ( 0.8%) | 29 ( 0.9%) | 41 ( 0.9%) |
|  | **Other Black** | 3(0.1%) | 2 ( 0.0%) | 2 ( 0.0%) | 6 ( 0.2%) | 5 ( 0.2%) | 9 ( 0.2%) |
|  | **Mixed** | 16(0.6%) | 39 ( 0.8%) | 29 ( 0.6%) | 63 ( 2.5%) | 77 ( 2.4%) | 119 ( 2.8%) |
|  | **Bangladeshi** | 10(0.4%) | 18 ( 0.4%) | 15 ( 0.3%) | 39 ( 1.5%) | 49 ( 1.5%) | 85 ( 2.0%) |
|  | **Indian** | 40(1.4%) | 105 ( 2.1%) | 87 ( 1.9%) | 62 ( 2.4%) | 79 ( 2.5%) | 130 ( 3.0%) |
|  | **Pakistani** | 19(0.7%) | 63 ( 1.3%) | 51 ( 1.1%) | 90 ( 3.5%) | 112 ( 3.5%) | 177 ( 4.1%) |
|  | **Chinese** | 5(0.2%) | 9 ( 0.2%) | 7 ( 0.2%) | 2 ( 0.1%) | 6 ( 0.2%) | 6 ( 0.1%) |
|  | **Other Asian** | 5(0.2%) | 25 ( 0.5%) | 20 ( 0.4%) | 19 ( 0.7%) | 24 ( 0.8%) | 31 ( 0.7%) |
|  | **Other** | 4(0.1%) | 24 ( 0.5%) | 15 ( 0.3%) | 5 ( 0.2%) | 10 ( 0.3%) | 20 ( 0.5%) |
| **Migrate N(%)** | **Lives in the UK** | 2829(100%) | 5462 (99.3%) | 5207 (99.3%) | 2017 (99.4%) | 2528 (99.4%) | 4441 (99.5%) |
|  | **Emigrated** | 0(0%) | 40 ( 0.7%) | 38 ( 0.7%) | 13 ( 0.6%) | 16 ( 0.6%) | 23 ( 0.5%) |
| **Education** | **University Higher** | 14 (18.2%) | 24 (12.6%) | 26 (14.1%) | 51 ( 3.9%) | 78 ( 4.8%) | 122 ( 4.6%) |
|  | **University** | 21 (27.3%) | 43 (22.5%) | 39 (21.1%) | 1112 (85.6%) | 1387 (84.5%) | 2230 (83.6%) |
|  | **Foundation** | - | - | - | 28 ( 2.2%) | 46 ( 2.8%) | 79 ( 3.0%) |
|  | **Other postgraduate** | 7 ( 9.1%) | 16 ( 8.4%) | 15 ( 8.1%) | 2 ( 0.2%) | 3 ( 0.2%) | 8 ( 0.3%) |
|  | **A AS S levels** | 6 ( 7.8%) | 19 ( 9.9%) | 18 ( 9.7%) |  |  |  |
|  | **A Level/A2 Level** | - | - | - | 18 ( 1.4%) | 19 ( 1.2%) | 39 ( 1.5%) |
|  | **AS Level** | - | - | - | 2 ( 0.2%) | 4 ( 0.2%) | 4 ( 0.1%) |
|  | **GCSE** |  |  |  | 2 ( 0.2%) | 5 ( 0.3%) | 11 ( 0.4%) |
|  | **GCSE grades D-G** | 5 ( 6.5%) | 10 ( 5.2%) | 11 ( 5.9%) | - | - | - |
|  | **O level GCSE grades A-C** | 16 (20.8%) | 50 (26.2%) | 38 (20.5%) | - | - | - |
|  | **Other academic** | 4 ( 5.2%) | 11 ( 5.8%) | 12 ( 6.5%) | 8 ( 0.6%) | 7 ( 0.4%) | 18 ( 0.7%) |
|  | **vocational qualifications** | - | - | - |  |  |  |
|  | **BTEC** | - | - | - | 23 ( 1.8%) | 31 ( 1.9%) | 46 ( 1.7%) |
|  | **GNVQ/NVQ/HNC** | - | - | - | 26 ( 2.0%) | 30 ( 1.8%) | 53 ( 2.0%) |
|  | **SQA** | - | - | - | 3 ( 0.2%) | 3 ( 0.2%) | 5 ( 0.2%) |
|  | **apprenticeship** | - | - | - | 0 ( 0.0%) | 1 ( 0.1%) | 3 ( 0.1%) |
|  | **City and Guilds Certificate** | - | - | - | 6 ( 0.5%) | 4 ( 0.2%) | 10 ( 0.4%) |
|  | **Vocational driving licence** | - | - | - | 0 | 0 | 0 |
|  | **Other qualification** | - | - | - | 18 ( 1.4%) | 23 ( 1.4%) | 40 ( 1.5%) |
|  | **None above** | 4 ( 5.2%) | 18 ( 9.4%) | 26 (14.1%) | - | - | - |
| **Occupation** | **Employed and Working** | 1539 (57.4%) | 3650 (68.2%) | 3365 (65.7%) | 240 (26.4%) | 472 (52.4%) | 786 (18.0%) |
|  | **Employed but on paid leave** | 416 (15.5%) | 149 ( 2.8%) | 311 ( 6.1%) | 338 (37.1%) | 32 ( 3.6%) | 231 ( 5.3%) |
|  | **Employed and on unpaid leave** | 15 ( 0.6%) | 26 ( 0.5%) | 25 ( 0.5%) | 34 ( 3.7%) | 8 ( 0.9%) | 26 ( 0.6%) |
|  | **Apprenticeship** | 1 ( 0.0%) | 2 ( 0.0%) | 1 ( 0.0%) | 50 ( 5.5%) | 60 ( 6.7%) | 150 ( 3.4%) |
|  | **voluntary work** | 13 ( 0.5%) | 26 ( 0.5%) | 20 ( 0.4%) | 2 ( 0.2%) | 11 ( 1.2%) | 22 ( 0.5%) |
|  | **Self-employed and working** | 162 ( 6.0%) | 535 (10.0%) | 468 ( 9.1%) | 6 ( 0.7%) | 18 ( 2.0%) | 32 ( 0.7%) |
|  | **Self-employed but not working** | 125 ( 4.7%) | 94 ( 1.8%) | 127 ( 2.5%) | 11 ( 1.2%) | 9 ( 1.0%) | 21 ( 0.5%) |
|  | **Unemployed** | 52 ( 1.9%) | 150 ( 2.8%) | 128 ( 2.5%) | 153 (16.8%) | 212 (23.6%) | 332 ( 7.6%) |
|  | **Permanently sick** | 31 ( 1.2%) | 101 ( 1.9%) | 111 ( 2.2%) | 6 ( 0.7%) | 16 ( 1.8%) | 21 ( 0.5%) |
|  | **Looking after home** | 209 ( 7.8%) | 333 ( 6.2%) | 293 ( 5.7%) | 11 ( 1.2%) | 15 ( 1.7%) | 29 ( 0.7%) |
|  | **In education** | 1 ( 0.0%) | 21 ( 0.4%) | 28 ( 0.5%) | 14 ( 1.5%) | 24 ( 2.7%) | 2677 (61.2%) |
|  | **Retired** | 89 ( 3.3%) | 202 ( 3.8%) | 210 ( 4.1%) | 0 ( 0.0%) | 1 ( 0.1%) | 1 ( 0.0%) |
|  | **Doing something else** | 30 ( 1.1%) | 65 ( 1.2%) | 33 ( 0.6%) | 45 ( 4.9%) | 22 ( 2.4%) | 49 ( 1.1%) |
| **Chronic illness?** | **Chronic illness n(%)** | 1316 (47.4%) | 1625 (47.3%) | 2592 (49.8%) | 834 (33.1%) | 561 (37.9%) | 1756 (40.1%) |
|  | **No chronic illness** | 1458 (52.6%) | 1812 (52.7%) | 2618 (50.2%) | 1687 (66.9%) | 920 (62.1%) | 2622 (59.9%) |
| **Relationship Status N(%)** | **First Married** | 1981 (70.0%) | 3460 (68.4%) | 3235 (68.7%) | - | - | - |
|  | **Second or later Married** | 279 ( 9.9%) | 535 (10.6%) | 504 (10.7%) | - | - | - |
|  | **Widowed** | 30 ( 1.1%) | 56 ( 1.1%) | 58 ( 1.2%) | - | - | - |
|  | **Divorced** | 244 ( 8.6%) | 430 ( 8.5%) | 378 ( 8.0%) | - | - | - |
|  | **Civil Partner** | 15 ( 0.5%) | 46 ( 0.9%) | 45 ( 1.0%) | - | - | - |
|  | **Don't Know** | 2 ( 0.1%) | 6 ( 0.1%) | 3 ( 0.1%) | - | - | - |
|  | **Don't want answer** | 0 ( 0.0%) | 5 ( 0.1%) | 4 ( 0.1%) | - | - | - |
|  | **Former civil partner** | 4 ( 0.1%) | 6 ( 0.1%) | 5 ( 0.1%) | - | - | - |
|  | **Legally separated** | 63 ( 2.2%) | 120 ( 2.4%) | 113 ( 2.4%) | - | - | - |
|  | **Single never** | 211 ( 7.5%) | 395 ( 7.8%) | 363 ( 7.7%) | - | - | - |
|  | **Surviving Civil Partner** | 0 ( 0.0%) | 1 ( 0.0%) | 1 ( 0.0%) | - | - | - |

## SI2: Number and proportion of respondents with each chronic illness

|  | **MCS Parents** | | | **MCS Cohort Members** | | |
| --- | --- | --- | --- | --- | --- | --- |
|  | **W1** | **W2** | **W3** | **W1** | **W2** | **W3** |
| **Cancer** | 50 (1.8%) | 34 (1.0%) | 61 (1.2%) | 2 (0.1%) | 1 (0.1%) | 2 (0.0%) |
| **Cystic fibrosis** | 0 ( 0.0%) | 0 ( 0.0%) | 1 (0.0%) | 1 (0.0%) | 0 (0.0%) | 1 (0.0%) |
| **Asthma** | 314 (11.3%) | 388 (11.3%) | 584 (11.2%) | 352 (14.0%) | 195 (13.1%) | 557 (12.6%) |
| **Chronic Obstructive Pulmonary Disease** | 15 (0.5%) | 20 (0.6%) | 35 (0.7%) | 1 (0.0%) | 1 (0.1%) | 5 (0.1%) |
| **Wheezy Bronchitis** | 9 (0.3%) | 10 (0.3%) | 15 (0.3%) | 3 (0.1%) | 4 (0.3%) | 6 (0.1%) |
| **Diabetes** | 107 (3.9%) | 165 (4.8%) | 258 (4.9%) | 19 (0.8%) | 9 (0.6%) | 31 (0.7%) |
| **Recurrent backache/prolapsed  disc/sciatica/other back problem** | 345 (12.4%) | 382 (11.1%) | 673 (12.9%) | 104 (4.1%) | 55 (3.7%) | 181 (4.1%) |
| **Problems with hearing** | 156 (5.6%) | 144 (4.2%) | 294 (5.6%) | 46 (1.8%) | 26 (1.7%) | 84 (1.9%) |
| **High blood pressure** | 333 (12.0%) | 396 (11.5%) | 630 (12.1%) | 12 (0.5%) | 7 (0.5%) | 33 (0.7%) |
| **Heart problems** | 51 (1.8%) | 59 (1.7%) | 108 (2.1%) | 11 (0.4%) | 5 (0.3%) | 19 (0.4%) |
| **Depression or other  emotional/nervous/psychiatric probs** | 233 (8.4%) | 432 (12.6%) | 697 (13.4%) | 412 (16.3%) | 368 (24.9%) | 1146 (26.2%) |
| **Obesity** | 284 (10.2%) | 445 (13.0%) | 735 (14.1%) | 50 (2.0%) | 53 (3.6%) | 169 (3.9%) |
| **Chronic obstructive airways disease** | 3 (0.1%) |  |  | 1 (0.0%) |  |  |
| **Infection** | 14 (0.5%) | 15 (0.4%) | 22 (0.4%) | 20 (0.8%) | 11 (0.7%) | 32 (0.7%) |
| **HIV or AIDs** | 15 (0.5%) | 10 (0.3%) | 26 (0.5%) | 8 (0.3%) | 0 (0.0%) | 10 (0.2%) |
| **Condition affecting the brain and nerves** | 35 (1.3%) | 42 (1.2%) | 66 (1.3%) | 12 (0.5%) | 8 (0.5%) | 26 (0.6%) |

## SI3: Descriptive statistics of the MCS and MCSP samples broken down by survey wave

|  | Millennium Cohort Study | | | | | Millennium Cohort Study Parent | | | | |
| --- | --- | --- | --- | --- | --- | --- | --- | --- | --- | --- |
|  | N | mean | sd | min | max | N | mean | sd | min | max |
| **Wave 0** |  |  |  |  |  |  |  |  |  |  |
| SWEMWBS | 5324 | 3.49 | 0.67 | 1 | 5 | NA |  |  |  |  |
| KESSLER | 5325 | 2.27 | 0.81 | 1 | 5 | 6554 | 1.63 | 0.65 | 1 | 5 |
| Social Provision | 4231 | 1.77 | 0.38 | 0 | 2 | NA |  |  |  |  |
| Has chronic ill. | 5274 | 0.18 | 0.38 | 0 | 1 | NA |  |  |  |  |
| **Wave 1** |  |  |  |  |  |  |  |  |  |  |
| SWEMWBS | 2328 | 3.29 | 0.69 | 1 | 5 | 2615 | 3.73 | 0.61 | 1 | 5 |
| KESSLER | 2341 | 2.40 | 0.86 | 1 | 5 | 2624 | 1.66 | 0.59 | 1 | 5 |
| Social Provision | 2349 | 1.80 | 0.35 | 0 | 2 | 2631 | 1.84 | 0.32 | 0 | 2 |
| Rely so if sick | 2347 | 3.65 | 0.68 | 1 | 4 | 2631 | 3.74 | 0.59 | 1 | 4 |
| Loneliness | 2346 | 1.77 | 0.57 | 1 | 3 | 2630 | 1.33 | 0.47 | 1 | 3 |
| Has chronic ill. | 2521 | 0.33 | 0.47 | 0 | 1 | 2774 | 0.47 | 0.50 | 0 | 1 |
| **Wave 2** |  |  |  |  |  |  |  |  |  |  |
| SWEMWBS | 2943 | 3.31 | 0.71 | 1 | 5 | 5222 | 3.64 | 0.65 | 1 | 5 |
| KESSLER | 2953 | 2.41 | 0.90 | 1 | 5 | 5237 | 1.68 | 0.69 | 1 | 5 |
| Social Provision | 2961 | 1.76 | 0.39 | 0 | 2 | 5250 | 1.76 | 0.40 | 0 | 2 |
| Rely so if sick | 2961 | 3.49 | 0.78 | 1 | 4 | 5251 | 3.61 | 0.71 | 1 | 4 |
| Loneliness | 2960 | 1.78 | 0.60 | 1 | 3 | 5250 | 1.37 | 0.50 | 1 | 3 |
| Has chronic ill. | 1483 | 0.38 | 0.49 | 0 | 1 | 3439 | 0.47 | 0.50 | 0 | 1 |
| **Wave 3** |  |  |  |  |  |  |  |  |  |  |
| SWEMWBS | 4273 | 3.20 | 0.70 | 1 | 5 | 4832 | 3.58 | 0.64 | 1 | 5 |
| KESSLER | 4279 | 2.43 | 0.93 | 1 | 5 | 4842 | 1.74 | 0.72 | 1 | 5 |
| Social Provision | 2545 | 1.74 | 0.40 | 0 | 2 | 3820 | 1.78 | 0.39 | 0 | 2 |
| Rely so if sick | 4272 | 3.55 | 0.77 | 1 | 4 | 4853 | 3.57 | 0.75 | 1 | 4 |
| Loneliness | 4281 | 1.80 | 0.60 | 1 | 3 | 4849 | 1.43 | 0.52 | 1 | 3 |
| Has chronic ill. | 4378 | 0.40 | 0.49 | 0 | 1 | 5210 | 0.50 | 0.50 | 0 | 1 |

## SI4: Full set of path coefficients for models in Figure 1: outcome SWEMWBS

|  | MCS Sample | | | | MCS Parent Sample | | | |
| --- | --- | --- | --- | --- | --- | --- | --- | --- |
|  | M1 | | M2 | | M3 | | M4 | |
| SWEMWBS 0 on | Coeff. | SE | Coeff. | SE | Coeff. | SE | Coeff. | SE |
| Chronic illness 0 | -.298** | .021 | -.284** | .021 |  |  |  |  |
| Social provision 0 |  |  | .461** | .015 |  |  |  |  |
| SWEMWBS 1 on |  |  |  |  |  |  |  |  |
| Chronic illness 1 | -.257** | .025 | -.146** | .022 | -.130** | .019 | -.058** | .018 |
| Can rely on so 1 |  |  | .039** | .007 |  |  | .079** | .007 |
| Social provision 1 |  |  | .461** | .015 |  |  | .219** | .014 |
| Loneliness 1 |  |  | -.423** | .011 |  |  | -.502** | .011 |
| SWEMWBS 2 on |  |  |  |  |  |  |  |  |
| Chronic illness 2 | -.345** | .025 | -.171** | .022 | -.212** | .017 | -.117** | .014 |
| Can rely on so 2 |  |  | .039** | .007 |  |  | .079** | .007 |
| Social provision 2 |  |  | .461** | .015 |  |  | .219** | .014 |
| Loneliness 2 |  |  | -.423** | .011 |  |  | -.502** | .011 |
| SWEMWBS 3 on |  |  |  |  |  |  |  |  |
| Chronic illness 3 | -.316** | .025 | -.164** | .016 | -.202** | .015 | -.118** | .013 |
| Can rely on so 3 |  |  | .039** | .007 |  |  | .079** | .007 |
| Social provision 3 |  |  | .461** | .015 |  |  | .219** | .014 |
| Loneliness 3 |  |  | -.423** | .011 |  |  | -.502** | .011 |
| Can rely on so 1 on |  |  |  |  |  |  |  |  |
| Chronic illness 1 |  |  | -.091** | .028 |  |  | -.083** | .020 |
| Can rely on so 2 on |  |  |  |  |  |  |  |  |
| Chronic illness 2 |  |  | -.146** | .031 |  |  | -.075** | .020 |
| Can rely on so 3 on |  |  |  |  |  |  |  |  |
| Chronic illness 3 |  |  | -.131** | .023 |  |  | -.069** | .019 |
| Social provision 0 on |  |  |  |  |  |  |  |  |
| Chronic illness 0 |  |  | -.059** | .014 |  |  |  |  |
| Social provision 1 on |  |  |  |  |  |  |  |  |
| Chronic illness 1 |  |  | -.060** | .013 |  |  | -.050** | .011 |
| Social provision 2 on |  |  |  |  |  |  |  |  |
| Chronic illness 2 |  |  | -.102** | .014 |  |  | -.051** | .011 |
| Social provision 3 on |  |  |  |  |  |  |  |  |
| Chronic illness 3 |  |  | -.094** | .013 |  |  | -.049** | .011 |
| Loneliness 1 on |  |  |  |  |  |  |  |  |
| Chronic illness 1 |  |  | .220** | .021 |  |  | .120** | .014 |
| Loneliness 2 on |  |  |  |  |  |  |  |  |
| Chronic illness 2 |  |  | .293** | .021 |  |  | .147** | .013 |
| Loneliness 3 on |  |  |  |  |  |  |  |  |
| Chronic illness 3 |  |  | .259** | .017 |  |  | .139** | .012 |
| N | 5552 | | 5552 | | 7479 | | 7479 | |
| Chi2(df) | 273.393 (12) | | 987.854 (79) | | 81.726 (6) | | 420.358 (48) | |
| CFI | .961 | | .959 | | .990 | | .985 | |
| RMSEA (90% CI) | .062 (.056-.069) | | .043 (.041-.046) | | .041 (.033-.049) | | .032 (.029-.035) | |

## SI5: Full set of path coefficients for models in Figure 1: outcome KESSLER

|  | MCS Sample | | | | MCS Parent Sample | | | |
| --- | --- | --- | --- | --- | --- | --- | --- | --- |
|  | M1 | | M2 | | M3 | | M4 | |
| KESSLER 0 on | Coeff. | SE | Coeff. | SE | Coeff. | SE | Coeff. | SE |
| Chronic illness 0/1 | .434** | .025 | .433** | .025 |  |  |  |  |
| Social provision 0 |  |  | -.469** | .018 |  |  |  |  |
| KESSLER 1 on |  |  |  |  |  |  |  |  |
| Chronic illness 1 | .393** | .029 | .289** | .026 | .224** | .018 | .164** | .016 |
| Can rely on so 1 |  |  | -.003 | .009 |  |  | -.045** | .007 |
| Social provision 1 |  |  | -.469** | .018 |  |  | -.082** | .014 |
| Loneliness 1 |  |  | .555** | .013 |  |  | .470** | .011 |
| KESSLER 2 on |  |  |  |  |  |  |  |  |
| Chronic illness 2 | .532** | .029 | .357** | .026 | .289** | .016 | .224** | .015 |
| Can rely on so 2 |  |  | -.003 | .009 |  |  | -.045** | .007 |
| Social provision 2 |  |  | -.469** | .018 |  |  | -.082** | .014 |
| Loneliness 2 |  |  | .555** | .013 |  |  | .470** | .011 |
| KESSLER 3 on |  |  |  |  |  |  |  |  |
| Chronic illness 3 | .512** | .023 | .367** | .020 | .283** | .016 | .222** | .014 |
| Can rely on so 3 |  |  | -.003 | .009 |  |  | -.045** | .007 |
| Social provision 3 |  |  | -.469** | .018 |  |  | -.082** | .014 |
| Loneliness 3 |  |  | .555** | .013 |  |  | .470** | .011 |
| Can rely on 1 on |  |  |  |  |  |  |  |  |
| Chronic illness 1 |  |  | -.090** | .028 |  |  | -.083** | .020 |
| Can rely on 2 on |  |  |  |  |  |  |  |  |
| Chronic illness 2 |  |  | -.139** | .031 |  |  | -.072** | .019 |
| Can rely on 3 on |  |  |  |  |  |  |  |  |
| Chronic illness 3 |  |  | -.128** | .023 |  |  | -.068** | .019 |
| Social provision 0 on |  |  |  |  |  |  |  |  |
| Chronic illness 0 |  |  | -.060** | .014 |  |  |  |  |
| Social provision 1 on |  |  |  |  |  |  |  |  |
| Chronic illness 1 |  |  | -.058** | .013 |  |  | -.051** | .011 |
| Social provision 2 on |  |  |  |  |  |  |  |  |
| Chronic illness 2 |  |  | -.094** | .014 |  |  | -.051** | .011 |
| Social provision 3 on |  |  |  |  |  |  |  |  |
| Chronic illness 3 |  |  | -.096** | .013 |  |  | -.049** | .011 |
| Loneliness 1 on |  |  |  |  |  |  |  |  |
| Chronic illness 1 |  |  | .219** | .021 |  |  | .121** | .014 |
| Loneliness 2 on |  |  |  |  |  |  |  |  |
| Chronic illness 2 |  |  | .277** | .021 |  |  | .143** | .013 |
| Loneliness 3 on |  |  |  |  |  |  |  |  |
| Chronic illness 3 |  |  | .255** | .017 |  |  | .137** | .012 |
| N | 5552 | | 5552 | | 7496 | | 7496 | |
| Chi2(df) | 515.763 (12) | | 1586.850 (79) | | 166.285 (8) | | 1670.899 (59) | |
| CFI | .942 | | .932 | | .986 | | .944 | |
| RMSEA | .087 (.081-.092) | | .059 (.056-.062) | | .051 (.045-.058) | | .060 (.058-.063) | |

Standard errors in parentheses

## SI6: Full set of parameters of models given in Table 2 in main manuscript

| Independent | Dependent | b | se | Dependent | b | se |
| --- | --- | --- | --- | --- | --- | --- |
| Specific sup W1 | WEMWBS W1 | 0.066** | (0.005) | KESSLER W1 | -0.041** | (0.006) |
| Soc prov W1 |  | 0.258** | (0.011) |  | -0.153** | (0.012) |
| Lonely W1 |  | -0.477** | (0.008) |  | 0.605** | (0.009) |
| Chronic (psy) W1 |  | -0.309** | (0.021) |  | 0.598** | (0.022) |
| Chronic (phy) W1 |  | -0.010 | (0.014) |  | 0.053** | (0.015) |
| MCS (vs MCSP) |  | -0.131** | (0.009) |  | 0.398** | (0.010) |
| Specific sup W2 | WEMWBS W2 | 0.066** | (0.005) | KESSLER W2 | -0.041** | (0.006) |
| Soc prov W2 |  | 0.258** | (0.011) |  | -0.153** | (0.012) |
| Lonely W2 |  | -0.477** | (0.008) |  | 0.605** | (0.009) |
| MCS (vs MCSP) |  | -0.131** | (0.009) |  | 0.398** | (0.010) |
| Chronic (psy) W2 |  | -0.335** | (0.017) |  | 0.666** | (0.018) |
| Chronic (phy) W2 |  | -0.054** | (0.010) |  | 0.070** | (0.014) |
| Specific sup W3 | WEMWBS W3 | 0.066** | (0.005) | KESSLER W3 | -0.041** | (0.006) |
| Soc prov W3 |  | 0.258** | (0.011) |  | -0.153** | (0.012) |
| Lonely W3 |  | -0.477** | (0.008) |  | 0.605** | (0.009) |
| MCS (vs MCSP) |  | -0.131** | (0.009) |  | 0.398** | (0.010) |
| Chronic (psy) W3 |  | -0.345** | (0.013) |  | 0.655** | (0.015) |
| Chronic (phy) W3 |  | -0.019+ | (0.010) |  | 0.065** | (0.012) |
| Chronic (psy) W1 | Spec. sup W1 | -0.250** | (0.026) | Spec. sup W1 | -0.251** | (0.026) |
| Chronic (phy) W1 |  | -0.042* | (0.018) |  | -0.050** | (0.018) |
| MCS (vs MCSP) |  | -0.050** | (0.011) |  | -0.048** | (0.012) |
| MCS (vs MCSP) | Spec. sup W2 | -0.050** | (0.011) | Spec. sup W2 | -0.048** | (0.012) |
| Chronic (psy) W2 |  | -0.301** | (0.024) |  | -0.293** | (0.023) |
| Chronic (phy) W2 |  | -0.046** | (0.013) |  | -0.032+ | (0.016) |
| MCS (vs MCSP) | Spec. sup W3 | -0.050** | (0.011) | Spec. sup W3 | -0.048** | (0.012) |
| Chronic (psy) W3 |  | -0.247** | (0.019) |  | -0.249** | (0.019) |
| Chronic (phy) W3 |  | -0.086** | (0.016) |  | -0.070** | (0.015) |
| Chronic (psy) W1 | Soc. prov. W1 | -0.164** | (0.014) | Soc. prov. W1 | -0.161** | (0.013) |
| Chronic (phy) W1 |  | -0.028** | (0.009) |  | -0.033** | (0.009) |
| MCS (vs MCSP) |  | -0.002 | (0.006) |  | 0.000 | (0.007) |
| MCS (vs MCSP) | Soc. prov. W2 | -0.002 | (0.006) | Soc. prov. W2 | 0.000 | (0.007) |
| Chronic (psy) W2 |  | -0.187** | (0.012) |  | -0.191** | (0.012) |
| Chronic (phy) W2 |  | -0.037** | (0.008) |  | -0.030** | (0.008) |
| MCS (vs MCSP) | Soc. prov. W3 | -0.002 | (0.006) | Soc. prov. W3 | 0.000 | (0.007) |
| Chronic (psy) W3 |  | -0.187** | (0.011) |  | -0.192** | (0.011) |
| Chronic (phy) W3 |  | -0.040** | (0.009) |  | -0.029** | (0.009) |
| Chronic (psy) W1 | Lonely W1 | 0.441** | (0.019) | Lonely W1 | 0.440** | (0.019) |
| Chronic (phy) W1 |  | 0.045** | (0.013) |  | 0.099** | (0.010) |
| MCS (vs MCSP) |  | 0.356** | (0.008) |  | 0.360** | (0.009) |
| Chronic (psy) W2 | Lonely W2 | 0.356** | (0.008) | Lonely W2 | 0.099** | (0.010) |
| MCS (vs MCSP) |  | 0.438** | (0.016) |  | 0.360** | (0.009) |
| Chronic (phy) W2 |  | 0.077** | (0.010) |  | 0.441** | (0.015) |
| Chronic (psy) W3 | Lonely W3 | 0.356** | (0.008) | Lonely W3 | 0.360** | (0.009) |
| MCS (vs MCSP) |  | 0.391** | (0.013) |  | 0.099** | (0.010) |
| Chronic (phy) W3 |  | 0.093** | (0.011) |  | 0.390** | (0.013) |
| *N* |  | 15250 |  |  | 15250 |  |

FIML estimated means of exposure

| mean(Chronic (psy) wave 1) | 0.131** | (0.004) |
| --- | --- | --- |
| mean(Chronic (phy) wave 1) | 0.332** | (0.006) |
| mean(mcs vs mcsp) | 0.508** | (0.004) |
| mean(Chronic (psy) wave 2) | 0.177** | (0.005) |
| mean(Chronic (phy) wave 2) | 0.314** | (0.006) |
| mean(Chronic (psy) wave 3) | 0.201** | (0.004) |
| mean(Chronic (phy) wave 3) | 0.332** | (0.005) |
